# Changes in peripheral lymphocyte populations in patients with advanced/recurrent ovarian cancer undergoing splenectomy during cytoreductive surgery

**DOI:** 10.1186/s13048-021-00860-7

**Published:** 2021-08-30

**Authors:** Wei Chen, Shuang Ye, Yutuan Wu, Xuan Pei, Libing Xiang, Bo Ping, Boer Shan, Huijuan Yang

**Affiliations:** 1 Department of Gynecologic Oncology, Fudan University Shanghai Cancer Center, Shanghai, China; 2 Department of Obstetrics and Gynecology, Minhang Hospital, Fudan University, The Central Hospital of Minhang District, Shanghai, China; 3 Department of Oncology, Shanghai Medical College, Fudan University, Shanghai, 200032 China; 4 Department of Pathology, Fudan University Shanghai Cancer Center, Shanghai, China

**Keywords:** Ovarian Neoplasms, Lymphocyte, Subpopulation, Flow cytometry, Splenectomy, Cytoreductive surgery

## Abstract

**Background:**

To investigate changes in peripheral lymphocyte subsets after splenectomy during cytoreductive surgery for advanced or recurrent ovarian cancers.

**Methods:**

We enrolled 83 patients with advanced or recurrent ovarian cancer who underwent cytoreductive surgery. Twenty patients who also underwent splenectomy were assigned to the splenectomy cohort and the rest were assigned to the non-splenectomy cohort. Flow cytometry was used to measure peripheral lymphocyte subsets consisting of T cells, regulatory T cells, natural killer cells, B cells, and activation antigens before and after surgery.

**Results:**

There was no difference in the number and distribution of peripheral lymphocyte subsets between the two cohorts before surgery. After surgery, we observed elevated levels of T cells (CD3^+^, CD3^+^CD8^+^) in the splenectomy cohort compared to those in the non-splenectomy cohort, and the difference was statistically significant. CD8^+^CD28^+^ T cells had a significant decreasing tendency (*P* = 0.011) while CD3^+^/HLA-DR^+^ T cells showed the opposite trend (*P* = 0.001) in the splenectomy cohort. The proportion of Tregs (*P* = 0.005) and B cells (*P* < 0.001) including CD3^−^/HLA-DR^+^ B cells (*P* = 0.007) increased after surgery, and the absolute number of T cells and NK cells decreased to different extents (*P* < 0.001) in the non-splenectomy cohort. The post-operative percentage of CD8^+^CD28^+^ T cells was less than the pre-operative percentage (*P* = 0.022), which was similar to the splenectomy cohort. There was no significant difference in progression-free survival or overall survival between the groups after a median follow-up time of 41 months.

**Conclusions:**

The changes in peripheral lymphocyte populations were different between patients with and those without splenectomy during cytoreductive surgery for ovarian cancers. T cells were increased and activated in the splenectomy cohort, whereas, B cells were increased and activated in the non-splenectomy cohort.

## Background

Ovarian cancer is the most lethal gynaecologic malignancy [1]. In China, more than 50,000 new cases, among which nearly half of the patients died, occurred in 2016, representing the second-highest cause of gynaecologic cancer-related death [2]. It is well accepted that optimal cytoreduction improves survival in ovarian cancer patients [3, 4]. In some circumstances, the removal of the spleen as part of upper abdominal surgery is required to achieve complete resection [5, 6].

As the spleen is one of the most important peripheral immune organs, splenectomy has been suggested to have an impact on immunological function [7]. Some studies found that people with splenectomy have a higher risk of developing overall cancer, as well as certain site-specific cancers [8, 9]. However, the spleen's antitumour function is bidirectional, which means that it has a positive antitumour role in the early stage of tumour formation and a negative immunological impact on tumour progression [10, 11]. Peripheral lymphocytes, which directly reflect a patient’s immune status, might be influenced by a variety of factors, including surgery, especially splenectomy. It is not clear whether splenectomy has a beneficial effect on immune function in advanced or recurrent ovarian cancer patients, beyond the benefit of reducing tumours.

In the present study, we aimed to investigate the effect of cytoreductive surgery with or without splenectomy on the immunological status of advanced and recurrent ovarian cancer patients. We compared the pre-and post-operative circulating lymphocyte distribution and analysed the changing trends of the two cohorts.

## Materials and methods

### Patient and comparison groups

The study was approved by the ethics committee of Fudan University Shanghai Cancer Center. All patients signed informed content. Splenectomy is performed to achieve optimal cytoreduction in patients with advanced or recurrent ovarian cancer with splenic metastasis. Patients who underwent optimal cytoreductive surgery for advanced or recurrent epithelial ovarian cancer between September 2016 and January 2019 were included. The staging was performed according to the criteria of the International Federation of Gynaecology and Obstetrics (FIGO) staging system (2014) [12]. Optimal cytoreductive surgery was defined as a residual tumour less than (or including) 1 cm after debulking surgery. Only patients who had undergone optimal cytoreduction involving upper abdominal surgery were eligible for inclusion. Of these, twenty patients underwent splenectomy during cytoreductive surgery for tumour involvement of the spleen. Other patients were grouped into the non- splenectomy group. Patients were excluded from this study if their FIGO stage was less than IIIC or they had non-epithelial ovarian cancer, the presence of other tumours, the presence of active infections or immune disease, or incomplete data.

Clinicopathological features were retrospectively abstracted from the electronic medical records, including age at first diagnosis in our institution, FIGO stage, histological subtypes, pre-treatment serum CA 125, residual disease, and adjuvant chemotherapy. Recurrent diseases were classified into platinum-sensitive disease if the interval time was > 6 months from the completion of the last platinum-based chemotherapy to the date of disease recurrence, and platinum-resistant disease if the interval time was < 6 months.

### Blood sample collection and flow cytometry

Written informed consent was acquired at admission. Peripheral blood samples were collected in heparinized tubes before treatment and after treatment. The average time interval from blood collection to surgery is three days while post-operative blood samples were also collected before discharge, usually one week after the operation. Blood cells were treated with red blood cell lysis buffer (Beyotime, China), labelled with fluorochrome-conjugated monoclonal antibodies and incubated at 4 °C for 20 min. Detailed flow procedures cytometry using BD Multitest 6-colour TBNK reagent (BD Pharmingen, USA) have been described in a previous publication from our institution [13].

### Statistical analysis

Statistical Package for Social Science (SPSS) software (Version 20.0, SPSS, Inc., Chicago, IL, USA) was used to analyse all data, and GraphPad Prism software (Version 6.0, GraphPad Software, Inc., La Jolla, CA, USA) was utilized to create the artwork. Continuous variables are presented as the mean ± standard deviation (SD), while categorical data arepresented as absolute numbers followed by percentages (%). Binomial data from two groups underwent chi-square analysis, and parametric data underwent independent samples t-tests to indentify any differences in characteristics. Lymphocyte subpopulations, that were non-normally distributed are shown as medians (quartile ranges, QRs). The difference between the groups was determined using the Mann–Whitney U test. Changes in lymphocyte subsets in patients undergoing different surgeries were evaluated using the Wilcoxon rank test. All *P* values reported were two-tailed, and a *P*-value < 0.05 was regarded as statistically significant.

## Results

### Patient characteristics

**Fig. 1:**
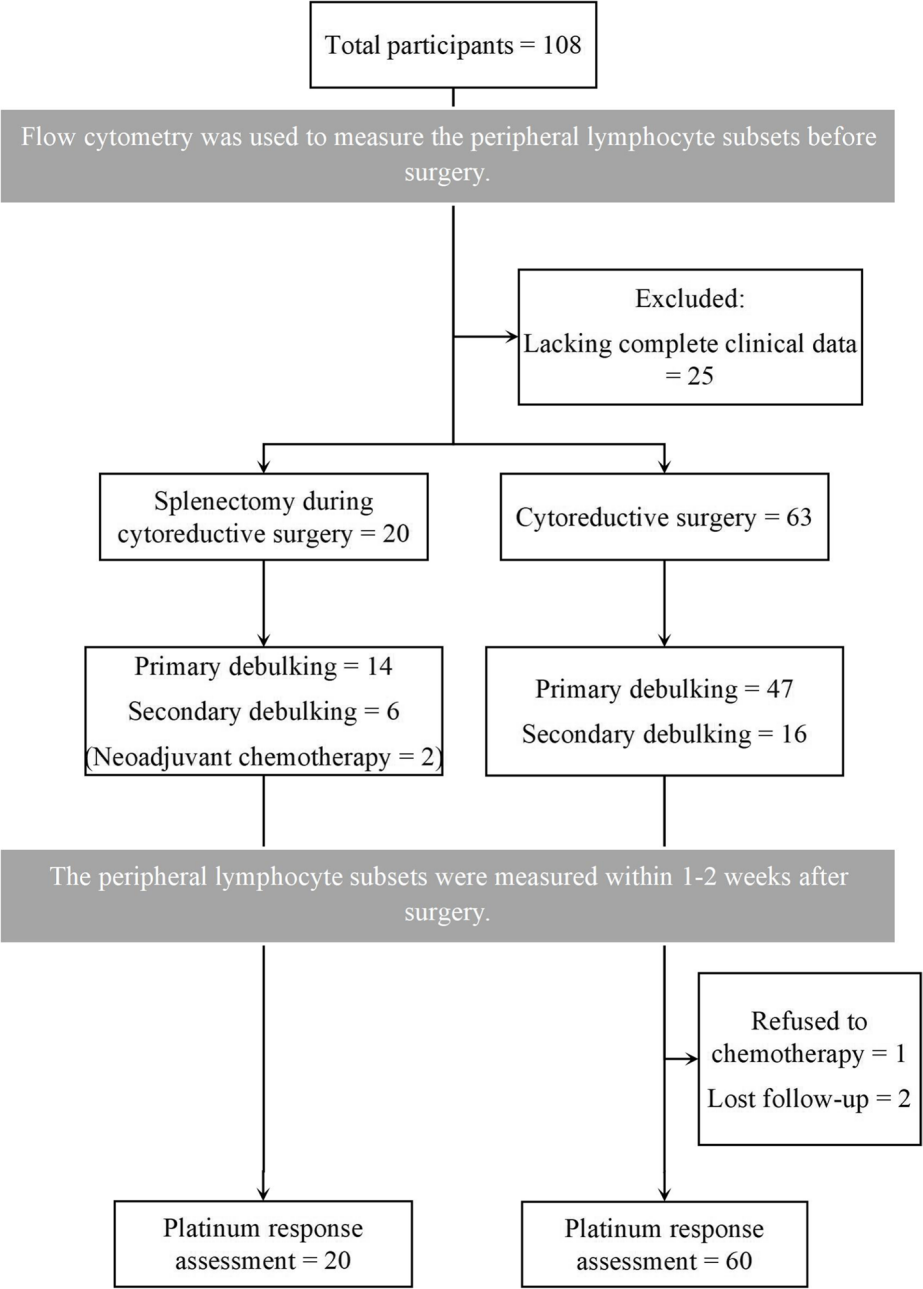
Schematic flowchart of the patients contained in the present study

During this period, a total of 108 patients received cytoreductive surgeries for advanced or recurrent ovarian cancer. Of them, 25 patients were excluded due to incomplete data. Among the remaining 83 patients, 20 patients were grouped into the splenectomy cohort (splenectomy during cytoreductive surgery) due to splenic metastasis, and 63 patients were grouped into the non-splenectomy cohort (cytoreductive surgery without splenectomy). At the time of analysis, all but 3 patients were available for platinum response assessment. The study participant flow diagram is shown in Fig. 1.

Table 1 shows the demographic and clinicopathological characteristics of the 83 patients. Primary diseases accounted for 70% and 67.5% of the patients in the splenectomy group and non-splenectomy group, respesctively. The age of the patients at the time of diagnosis and the level of CA125 before treatment were not significantly different between the two groups. Most of the patients had high-grade serous carcinomas (90% and 87.5%). Two patients in the splenectomy group received neoadjuvant chemotherapy before surgery, but no significant difference was noted (*P* = 0.056). All patients received optimal debulking for residual tumours smaller than 1 cm, while more than 60% of the patients achieved complete resection (65% in the splenectomy group vs. 63.5% in the non-splenectomy group). As shown in Fig. 1, 80 patients were available for platinum response assessment. Of them, platinum-resistant recurrence accounted for 30.0% in the splenectomy group and 14.3% in the non- splenectomy group, the difference was not statistically significant.

**Table 1:** Demographic and clinicopathological information (*n *= 83)

Variables	Splenectomy (*n* = 20)	Non-splenectomy (*n* = 63)	*P*-value
Age (years), mean (SD, 95% CI)	58 (9.7, 53–62)	54 (8.8, 52–56)	0.114^*^
Neoadjuvant chemotherapy (%)	2 (10%)	0	0.056^#^
Histology			
High-grade serous carcinoma	18 (90.0%)	55 (87.3%)	0.246^#^
Clear cell carcinoma	0	5 (7.9%)	
Endometrioid	1 (5.0%)	1 (1.6%)	
Carcinosarcoma	1 (5.0%)	0	
Low-grade serous carcinoma	0	2 (3.2%)	
Pre-treatment CA125 (U/mL), media (range)	718.9 (8.9–5000) ^a^	539.0 (8.3–5000)	0.446^**^
FIGO stage (%)			
IIIC	11 (55.0%)	33 (52.4%)	0.838^#^
IV	3 (15.0%)	14 (22.2%)	
Recurrence	6 (30.0%)	16 (25.4%)	
Residual disease (%)			
0	13 (65.0%)	40 (63.5%)	0.903^#^
≤ 1 cm (optimal)	7 (35.0%)	23 (36.5%)	
Platinum response (%) ^b^			
Sensitive	14 (70.0%)	51 (81.0%)	0.185^#^
Resistant	6 (30.0%)	9 (14.3%)	

*P*-value < 0.05 was deemed statistically significant

*Abbreviations*: *FIGO*   The International Federation of Gynecology and Obstetrics, *CA125* Cancer Antigen 125

^a^ The upper limit of CA 125 detection is 5000

^b^ Sixteen patients in the splenectomy group and 40 patients in the control group were available for platinum response assessment

^*^ Independent-Sample T-test

^**^ Mann–Whitney U test

^#^ chi-square or Fisher’s exact test

### Lymphocyte subsets in peripheral blood: Before surgery vs. after surgery

**Fig. 2:**
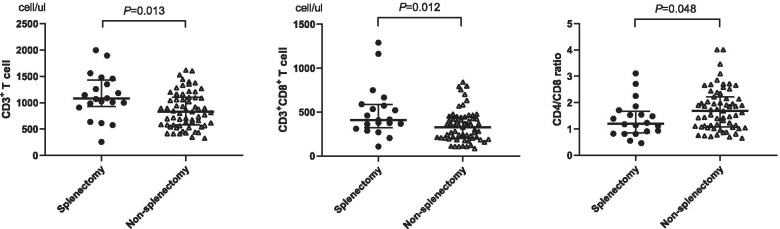
Comparisons with the statistical significance of circulating lymphocyte subsets after surgery (splenectomy vs. non-splenectomy)

By flow cytometry, the circulating lymphocyte subpopulations were measured, including T cells (CD3^+^), B cells (CD3^−^CD19^+^), and natural killer (NK) cells (CD3^−^CD16^+^56^+^). T cells consist of helper T cells (Th, CD3^+^CD4^+^), cytotoxic T cells (Tc, CD3^+^CD8^+^ including CD8^+^CD28^+^), and regulatory T cells (Tregs, CD4^+^CD25^+^CD127^−^). Activated T cells are mostly CD3^+^/HLA-DR^+^, and activated B cells and activated NK cells are mostly CD3^−^/HLA-DR^+^. The pre-operative and post-operative data of the two cohorts are presented in Table 2. Before surgery, the percentage and absolute number of peripheral lymphocyte subpopulations were quite comparable between the splenectomy cohort and the non- splenectomy cohort. After surgery, the difference between the two groups is shown in Fig. 2. The median absolute number of CD3^+^ T cells was significantly higher in the splenectomy cohort than in the non- splenectomy cohort (1079 vs. 821, *P* = 0.013). This was mainly due to the difference in the absolute number of CD3^+^CD8^+^ T cells (splenectomy vs. non- splenectomy: 411 vs. 313, *P* = 0.012). Therefore, the CD4/CD8 ratio of the splenectomy group was lower than that of the non-splenectomy group (1.2 vs. 1.7, *P* = 0.048).

**Table 2:** Distribution of peripheral lymphocyte subpopulations in the two cohorts before and after surgery

Peripheral lymphocyte subsets	Splenectomy group			Non-splenectomy group			*P*_1_	*P*_2_
	pre	post	*P*_3_	pre	post	*P*_4_		
Percentage (%)								
T cells (CD3^+^)	67.8 (17.3)	68.9 (16.0)	0.241	67.6 (14.48)	69.1 (12.9)	0.276	0.680	0.431
CD3^+^CD8^+^	30.1 (12.2)	26.2 (16.5)	0.210	25.2 (12.50)	27.1 (14.5)	0.056	0.109	0.289
CD8^+^CD28^+^	8.9 (4.1)	7.6 (3.9)	***0.011***	6.9 (4.3)	6.8 (3.4)	***0.022***	0.116	0.606
CD3^+^CD4^+^	37.7 (7.1)	35.5 (9.2)	0.768	38.2 (12.8)	39.7 (12.8)	0.292	0.616	0.070
Tregs (CD4^+^CD25^+^CD127^low^)	13.2 (3.3)	14.6 (4.1)	0.344	12.8 (4.0)	13.2 (4.2)	***0.005***	0.477	0.461
CD4/CD8	1.2 (0.8)	1.2 (0.8)	0.746	1.6 (0.9)	1.7 (1.3)	***0.038***	0.125	***0.048***
NK cells (CD3^−^CD16^+^56^+^)	16.1 (12.9)	11.2 (13.9)	0.312	15.8 (14.0)	13.5 (13.3)	***0.043***	0.721	0.563
B cells (CD3^−^CD19^+^)	10.5 (9.5)	11.4 (8.6)	0.922	11.3 (6.9)	13.9 (7.7)	** < *0.001***	0.880	0.195
HLA-DR^+^	22.5 (13.6)	26.2 (19.1)	***0.011***	22.8 (18.2)	26.3 (15.8)	***0.003***	0.812	0.864
CD3^+^/HLA-DR^+^	6.3 (17.4)	9.3 (19.4)	***0.001***	11.3 (13.9)	11.3 (14.6)	0.198	0.563	0.969
CD3^−^/HLA-DR^+^	10.3 (8.5)	13.7 (11.2)	0.177	11.6 (6.8)	13.6 (6.3)	***0.010***	0.570	0.843
CD3^+^/HLA-DR^−^	59.2 (17.3)	49.6 (22.6)	** < *0.001***	57.4 (17.2)	55.9 (18.6)	***0.002***	0.669	0.093
Absolute number (cell/ul)								
T cells (CD3^+^)	942 (522)	1079 (502)	0.522	1101 (466)	821 (543)	** < *0.001***	0.226	***0.013***
CD3^+^CD8^+^	406 (290)	411 (261)	0.709	416 (251)	313 (237)	** < *0.001***	0.868	***0.012***
CD3^+^CD4^+^	495 (273)	611 (263)	0.490	594 (416)	465 (311)	** < *0.001***	0.111	0.140
NK cells (CD3^−^CD16^+^56^+^)	201 (47)	161 (276)	0.595	255 (236)	162 (173)	** < *0.001***	0.250	0.510
B cells (CD3^−^CD19^+^)	152 (194)	149 (158)	0.241	164 (133)	160 (147)	0.380	0.572	0.570

*P*_2_ = Splenectomy vs. Control after surgery; *P*_3_ = Preoperative vs. Postoperative in the splenectomy cohort; *P*_4_ = Preoperative vs. Postoperative in the control cohort

^a^ Numbers were presented as median (quartile range)

^b^ Pre = before surgery; Post = after surgery

^c^*P* values with statistical significance were denoted. *P*_1_ = Splenectomy vs. Control before surgery

### Change trends before and after surgery: Splenectomy vs. non- splenectomy group

**Fig. 3:**
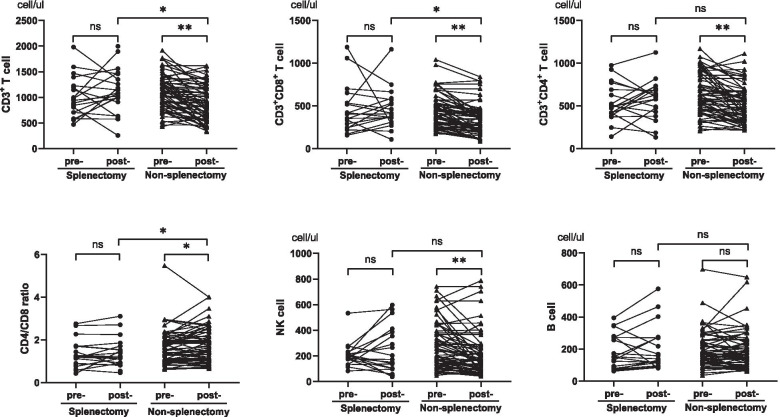
Changes in peripheral lymphocyte absolute numbers in the two cohorts after upfront surgery. Abbreviations: Pre- = before surgery; Post- = after surgery. ns = not significant; *, *P* < 0.05; ** *P* < 0.01

As shown in Fig. 3, in the splenectomy cohort, the median percentage of CD8^+^CD28^+^ T cell levels significantly decreased after the operation (pre-operative vs. post-operative: 8.9 vs. 7.6, *P* = 0.011), while the median percentage of activated antigens significantly increased (HLA-DR^+^, pre-operative vs. post-operative: 22.5 vs. 26.2, *P* = 0.011), which was mainly reflected in the increase in the percentage of activated CD3^+^/HLA-DR^+^ T cells (pre-operative vs. post-operative: 6.3 vs. 9.3, *P* = 0.001), with the decline in the percentage of CD3^+^/HLA-DR^−^ (pre-operative vs. post-operative: 59.2 vs. 49.6, *P* < 0.001). Other variables remained unchanged before and after the operation.

**Fig. 4:**
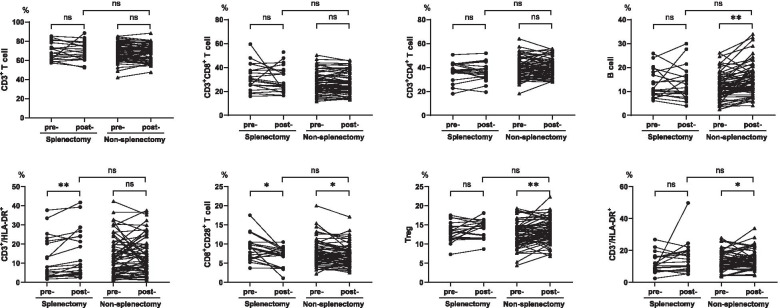
Changes in peripheral lymphocyte distribution in the two cohorts after upfront surgery. Abbreviations: Pre- = before surgery; Post- = after surgery. NS = not significant; *, *P* < 0.05; ** *P* < 0.01

Correspondingly, in addition to the percentage of T cells, almost all other variables changed significantly after debulking in the non-splenectomy cohort. The absolute numbers (presented in Fig. 4) of post-operative T cells (CD3^+^, CD3^+^CD8^+^, CD3^+^CD4^+^) were lower than those of pre-operative T cells (*P* < 0.001). The absolute number and percentage of post-operative NK cells (CD3^−^CD16^+^56^+^) also declined (absolute number: 255 vs. 162, *P* < 0.001; percentage: 15.8 vs. 13.5, *P* = 0.043). There were no significant differences in the absolute number of B cells (CD3^−^CD19^+^), but the percentage was higher than that before surgery (*P* < 0.001) which was mainly due to decreased absolute numbers of T cells and NK cells. The post-operative percentage of Tregs (CD4^+^CD25^+^CD127^low^) was significantly greater than the pre-operative percentage (*P* = 0.005). The post-operative percentage of CD8^+^CD28^+^ T cells decreased (*P* = 0.022), and the activation antigens (HLA^−^DR^+^) increased (*P* = 0.003).

In terms of absolute numbers of peripheral lymphocyte subpopulations, the changes in B cells were not significant in the two cohorts, while the NK cells decreased, in which the non- splenectomy cohort showed significant changes (*P* < 0.001). The post-operative T cells' value in the splenectomy group increased (*P* > 0.05), while that in the non-splenectomy group decreased (*P* < 0.01). In terms of the percentage, elevated levels of B cells and Tregs as declining levels of NK cells were found in both groups. These changes in the non-splenectomy group were significant (*P* < 0.05) and more obvious than those in the splenectomy group. CD8^+^CD28^+^ T cell levels in both cohorts decreased significantly (*P* < 0.05), but only the CD4/CD8 ratio of the non-splenectomy cohort increased (*P* = 0.038). After debulking, the proportion of lymphocytes with activated antigens (HLA-DR^+^) was greater than before in both groups. The changes were reflected in a significant increase in the percentage of CD3^+^/HLA-DR^+^ (activated T cells) in the splenectomy cohort (*P* = 0.001), compared with CD3^−^/HLA-DR^+^ in the non-splenectomy cohort (*P* = 0.007). Since the absolute number and proportion of NK cells in the non-splenectomy cohort decreased after surgery, we believed that the increased CD3^−^/HLA-DR^+^ was mostly composed of activated B cells.

### Follow-up of the recovery or progression of the disease

**Fig. 5:**
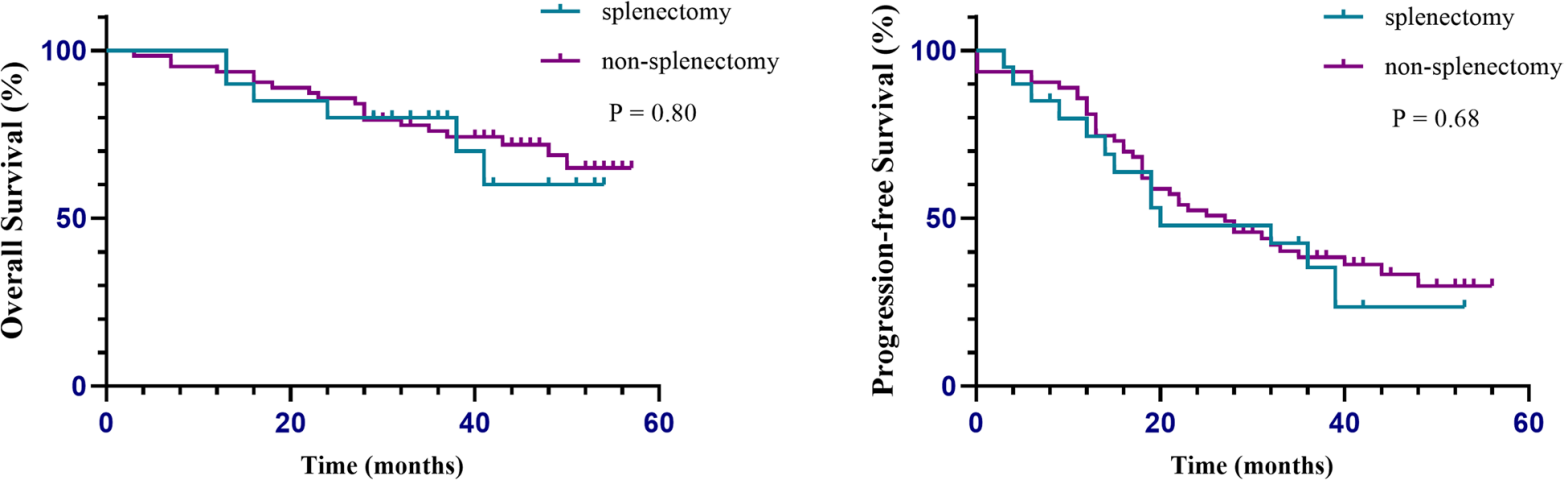
Kaplan–Meier analysis of overall survival and progression-free survival in patients who underwent splenectomy (blue) or non-splenectomy (purple) during cytoreductive surgery

After a median follow-up time of 41 months (range, 3–57) after surgery, 25 patients (30.1%) died from the disease. Thirty-one patients (37.3%) were still alive with the disease while 27 patients (32.5%) remained disease-free. For the group as a whole, the median PFS was 23 months (range, 0–56). The median PFS was 20 months in the splenectomy group and 27 months in the non-splenectomy group. As shown in Fig. 5, there were no statistically significant differences in PFS and OS between the two groups.

In summary, the changes in peripheral lymphocyte subpopulations were not completely identical in patients with splenectomy and those without splenectomy during debulking surgeries for advanced or recurrent ovarian cancers. CD3^+^ T cells and CD8^+^ T cells were significantly increased in patients with splenectomy, but decreased in patients without splenectomy. Activation antigen (HLA-DR^+^) was increased in T cells from patients with splenectomy and in B cells from patients without splenectomy. In the non-splenectomy group, NK cells significantly decreased and Tregs significantly increased after surgery, which did not reach statistical significance in the splenectomy group. CD8^+^CD28^+^ T cells were decreased in both groups. B cells were not significantly changed after surgery in either group.

## Discussion

It has been well accepted that patients with advanced tumours present an immunosuppressive status [14]. The spleen, as the most important peripheral immune organ, plays a negative immunological role [7]. Surgery is the standard treatment for OC and may assist in restoring the immune response [15]. A report suggested that the antitumour effect of the immunological function of the spleen was restored when the negative factor resulting in immune suppression produced by tumour cells was eliminated by debulking surgery [11]. Our results showed that the decrease in post-operative CD8^+^CD28^+^ T cells and NK cells in both groups indicated that the post-operative antitumour immunity of ovarian cancer patients was suppressed. Immune functional changes after surgery were less apparent in ovarian cancer patients with splenectomy than in those without splenectomy. Changes, such as a decrease in the absolute number of T cells and NK cells, and an increase in the proportion of Tregs (CD4^+^CD25^+^) and B cells, did not occur in the splenectomy group. Correspondingly, T cell levels of CD3^+^ and CD3^+^CD8^+^ in the non-splenectomy group were significantly lower than those in the splenectomy group. All these findings might suggest that the spleen played a part in post-operative immunosuppression in mediating the changes in peripheral blood lymphocytes. Therefore, splenectomy could reduce the immune suppression of ovarian cancer patients caused by surgery.

Increased circulating Tregs have been reported in patients with different kinds of malignant tumours, including ovarian cancer [13, 16–18]. Studies have considered that Tregs are mainly stored in the spleen [19]. They might be set free by the spleen into circulation during post-operative immune rearrangement, which could explain why CD4^+^ Tregs increased in patients with spleen preservation after surgery. CD4^+^ Tregs, one of the major Treg subtypes reported to date, can inhibit the tumour-specific T cell-mediated immune response and contribute to tumour escape and poor survival [20, 21].

The role of B cells in ovarian cancer is difficult to determine. An experiment in the laboratory indicated that B cells migrated to the site of the tumour and acquired the expression of immunosuppressive ligands and/or cytokines that contributed to the inhibition of the antitumour immune response [22]. Another publication showed that B cells infiltrating high-grade serous ovarian cancer omental metastases supported the development of an antitumour response [23]. The antitumour effect of B cells in peripheral circulation has rarely been studied. In our study, the reason for the increased B cell proportions may be the same as that for Tregs, since the spleen is the largest B cell-producing organ. The spleen might play a role in regulating the number of B cells in peripheral blood and remain stable when the depression of the immune response is evoked owing to serious attacks from surgical trauma. Therefore, the immune benefit is limited because the increase in the B cell proportion is based on the decrease in the T and NK cell proportions. The absolute number did not increase.

In the splenectomy group, the changes in immune status were not as obvious as those in the non-splenectomy group. We noted that the proportion of activated antigens (HLA-DR^+^) increased within 1–2 weeks post-operatively. It was taken into account in T cells (CD3^+^/HLA-DR^+^) of the splenectomy group and B cells (CD3^−^/HLA-DR^+^) of the non-splenectomy group. Studies on the role of HLA-DR^+^ in tumour immunity are rare and remain controversial. Our previous related study identified that an HLA-DR Treg population in the peripheral blood was significantly increased in cervical cancer patients compared to precancer patients and healthy donors [24]. Activated T cells (CD3^+^/HLA-DR^+^) were found to increase during chemotherapy in non-Hodgkin’s lymphoma patients and then decrease after chemotherapy, and elevated levels were associated with recurrence [25]. For ovarian cancer patients, activated T cells have been shown to be related to the size of residual tumours [26]. Under normal conditions, the majority of T cells are CD4^+^ T helper (Th) 1 and CD8^+^ T cytotoxic (Tc)1 cells. Th1/Tc1 immune responses, which enhance HLA-DR expression in T cells in inflamed sites, can lead to an increase in CD3^+^HLA-DR^+^ extracellular vesicle levels in the circulation [27]. Recent progress in cancer immunotherapy has revealed that Th1/Tc1 responses are suppressed in cancer patients and are restored by the blockade of the immune checkpoint receptor PD-1 or PD-L1 [28]. In our study, post-operative activated T cell elevation was unique to the splenectomy group. Regardless of whether this phenomenon was caused by post-operative inflammatory stimulation or decreased tumour load, it seemed to provide diverse ideas for post-operative immunotherapy. Kikuchi et.al found that the percentage and absolute number of CD3^−^/HLA-DR^+^ (B cells) in the peripheral blood of patients with advanced ovarian carcinoma were significantly lower than values from patients with benign ovarian tumours [26]. In the present study, we found an increase in CD3^−^/HLA-DR^+^ cells post-operatively only in the spleen-preserving group. Whether the increased activation of B cells can promote tumour immunity remains to be further studied.

Thus, the changes in peripheral lymphocyte populations were different between patients with and those without splenectomy during cytoreductive surgery for ovarian cancers. Prehn [29] concluded from the literature that small proportions of spleen cells usually stimulate tumour growth, in which case splenectomy is inhibitory. Larger proportions of the same cells usually inhibit tumour growth, in which case splenectomy results in tumour stimulation. It follows from this that splenectomy for optimal cytoreduction in more biologically aggressive diseases, such as advanced ovarian cancer should help to inhibit tumour growth. According to our data, T cells were increased and activated in the splenectomy cohort, whereas, B cells were increased and activated in the non-splenectomy cohort. The release of spleen function after surgery does not necessarily reflect removing the immunosuppression but seems to intensify tumour immune escape within two weeks after surgery. Ovarian cancer debulking combined with splenectomy has a slightly negative effect on peripheral lymphocyte subpopulation distribution but seems to be beneficial to the balance of T cell subsets and improves the antitumour immune function. Moreover, Rab et al. found that lymphocyte subsets are increased and persist over time in patients with splenectomy [30]. However, in terms of recurrence and survival data, quantitative changes in circulating lymphocyte subsets after splenectomy do not seem to play a significant role in long-term prognosis in patients receiving standard chemotherapy after splenectomy. In addition, McCann et al. found that patients whose disease was optimally cytoreduced had a worse median overall survival in the splenectomy group than in the non-splenectomy group (30 vs. 45 months, *P* < 0.045) [31]. Ugel et al. showed that the spleen is fundamentally important for tumour-induced tolerance, and splenectomy restores lymphocyte function and induces tumour regression when coupled with immunotherapy [32]. Our data, combined with this evidence, raise questions about whether the post-operative immune advantage in patients with splenectomy is compensated for over time. Can immunotherapy initiated as soon as possible after surgery improve the survival of these patients? These questions need to be addressed in future prospective trials.

The study has several limitations. First, a one-time snapshot of lymphocyte values could not reflect the kinetics. Prolonged observation might lead to a different conclusion considering the time needed for immune function to reach a different balance. We intend to collect consecutive blood samples for further study. Second, we analysed only the lymphocyte subsets mentioned above, and additional tests were required for other subsets. Third, only the preliminary work of flow cytometry has been carried out at present. Our future research will focus on further molecular support and mechanistic exploration to show the observed pattern. Last, given the limited case number, the result might partly explain the insignificant differences between ovarian cancer patients with and without splenectomy.

## Conclusion

The changes in peripheral lymphocyte populations were different between patients with and without splenectomy during cytoreductive surgery for ovarian cancers. T cells were increased and activated in the splenectomy cohort, whereas, B cells were increased and activated in the non-splenectomy cohort. Therefore, post-operative adjuvant therapy, including immunotherapy, needs to be customized.

## Data Availability

The datasets generated during and/or analyzed during the current study are available from the corresponding author on reasonable request.

## Abbreviations

Tregs: Regulatory T cells
NK: Natural killer
FIGO: The International Federation of Gynecology and Obstetrics
CA: Cancer antigen
